# Spatial Distribution Pattern of Wild Snow Leopard (*Panthera uncia*) Habitats in the Chengdu Section of the Giant Panda National Park

**DOI:** 10.3390/biology15050401

**Published:** 2026-02-28

**Authors:** Haipeng Zheng, Qinxi Hou, Zhidi Wang, Wanju Feng, Shiyao Wang, Qiujie Li, Jingjing Shuai, Peijun Ye, Chaowen Wang, Zhisong Yang, Hai Hu, Ke He

**Affiliations:** 1 Sichuan Academy of Giant Panda, Chengdu 610081, China; zhenghp2015@163.com (H.Z.); houqinxi@outlook.com (Q.H.); zhidi_wang@163.com (Z.W.); 19828320401@163.com (W.F.); yangzhisong@126.com (Z.Y.); 2 Chengdu Management Branch of Giant Panda National Park, Chengdu 610096, China; 13558884955@163.com; 3 Chengdu Academy of Agriculture and Forestry Sciences, Chengdu 610095, China; 15283179198@163.com; 4 Chengdu Nature Reserve and Wildlife Conservation Center, Chengdu 610036, China; sjj_404@163.com; 5 Chengdu Peoples Park, Chengdu 610015, China; yepeijun318@163.com (P.Y.); wcw102137@163.com (C.W.); 6 Sichuan Forestry and Grassland Survey and Planning Institute, Chengdu 610084, China

**Keywords:** *Panthera uncia*, Chengdu Area of the Giant Panda National Park, MaxEnt model, habitat

## Abstract

Snow leopards (*Panthera uncia*) are a flagship species for global biodiversity conservation, and their effective protection relies on accurate habitat assessment. This study focused on the Chengdu section of the Giant Panda National Park (Pengzhou, Dujiangyan, Chongzhou, Dayi), integrating terrain, climate, vegetation and human disturbance factors. Using the MaxEnt model (AUC = 0.943) and field infrared camera data, we evaluated snow leopard habitat quality. Results showed that: (1) 95.7% of snow leopard records were concentrated in Dayi County; (2) Key drivers included annual mean temperature (peak at −2 °C), annual mean ground temperature (peak at −1 °C) and human population density (>5 km), while NDVI (≈2000) had a significant negative effect; (3) Suitable habitat was 320.98 km^2^ (22.20%), decreasing from Qionglai Mountain to Minshan. This study fills regional survey gaps and provides a scientific basis for snow leopard conservation.

## Simple Summary

This study evaluates the habitat suitability of the vulnerable snow leopard (*Panthera uncia*) in the Chengdu section of the Giant Panda National Park, China, by integrating infrared camera surveys and MaxEnt modeling. The results reveal a highly restricted and uneven habitat distribution, with the core area concentrated in Dayi. Model analysis identified low annual ground temperature and significant avoidance of human settlements as the primary factors shaping habitat suitability. The findings underscore the urgent need to prioritize conservation efforts in the Dayi core area and implement targeted measures to mitigate human disturbance. This research provides critical scientific evidence to inform effective protection and management strategies for this flagship species in the region.

## 1. Introduction

As the spatial foundation upon which organisms depend for survival and reproduction, a habitat supports the environmental resources essential for their normal life processes [1]. Its quality not only influences the distribution and dispersal of species but also determines the structure and function of ecosystems. Consequently, protecting species’ habitats contributes to maintaining ecosystem stability and promotes the sustainable development of biodiversity [2]. Research on habitat suitability can delineate a species’ distributional range within a region, thereby enhancing our understanding of its distribution patterns, which is crucial for conservation monitoring [3]. However, drastically changing global climates and expanding human activities have led to significant habitat contraction for numerous species. It has been reported that the distribution ranges of 173 mammal species globally have shrunk by an average of 68% since the Industrial Revolution, with habitat loss reaching as high as 83% in Southeast Asia [4]. This range contraction is often accompanied by habitat loss and fragmentation, which impedes free species dispersal and increases the extinction risk for small, isolated populations [5]. This trend is also prevalent in alpine ecosystems, where it potentially impacts ecological structure and function, as well as species survival and reproduction [6].

The snow leopard (*Panthera uncia*), a flagship species and apex predator in alpine ecosystems, serves as an indicator of ecosystem health and integrity. Currently, the global wild population of snow leopards is estimated at fewer than 9000 individuals, with a habitat area of less than 3 million square kilometers [7]. It is listed as Vulnerable (VU) on the IUCN Red List of Threatened Species [8]. Approximately 62.5% of the global snow leopard population is concentrated in China, inhabiting high-altitude snowfield regions between 2500 and 5500 m in Tibet, Sichuan, Xinjiang, Qinghai, Gansu, Ningxia, and Inner Mongolia [9]. In China, it is classified as a National Class I Protected Animal [10]. It is estimated that only about 2% of snow leopard habitat in China has been effectively surveyed [11]. Current research on snow leopards in China has primarily focused on regions such as the Tianshan Mountains [12], the Three-River-Source Region [13,14], the Qilian Mountains [15,16], the Himalayan Mountains [17], and Western Sichuan [18,19]. The snow leopard population in Sichuan represents the eastern edge of the species’ distribution in China. Characterized by complex, diverse terrain and a mild, humid climate, its habitat exhibits certain differences from typical plateau ecosystems. To date, habitat studies on snow leopards in Sichuan have mainly concentrated on the Gongga Mountain region and the Wolong National Nature Reserve within the Qionglai Mountain range [19,20,21]. However, a systematic assessment of snow leopard habitats in Sichuan remains lacking. Notably, another significant ecological region in Sichuan—the Chengdu section of the Giant Panda National Park (hereafter referred to as the Chengdu section)—has not received sufficient research attention. As the world’s only national park located within a megacity [22], the Chengdu section bears the dual flagship species conservation mission for both the giant panda and the snow leopard. Since the first recorded distribution of snow leopards in this area in 2009 [23], their presence has also been confirmed in the Anzihe and Heishuihe Nature Reserves. Nevertheless, research on snow leopards in this region remains fragmented, lacking a comprehensive understanding of the population status and habitat ecology.

Therefore, this study aims to systematically investigate the population status, habitat distribution patterns, and habitat quality of snow leopards across the four regions (Pengzhou, Dujiangyan, Chongzhou, and Dayi) of the Giant Panda National Park Chengdu section. It further seeks to analyze the primary disturbance factors affecting snow leopard survival. By integrating field survey techniques such as infrared camera trapping with the MaxEnt species distribution model, this research seeks to address the aforementioned knowledge gaps, thereby providing scientific support for snow leopard conservation and habitat management. This study will not only fill the research void regarding snow leopard habitats in Sichuan Province but will also offer crucial data support and a theoretical foundation for the conservation and habitat management of snow leopard populations in Sichuan and across China.

## 2. Materials and Methods

### 2.1. Study Area

**Figure 1:**
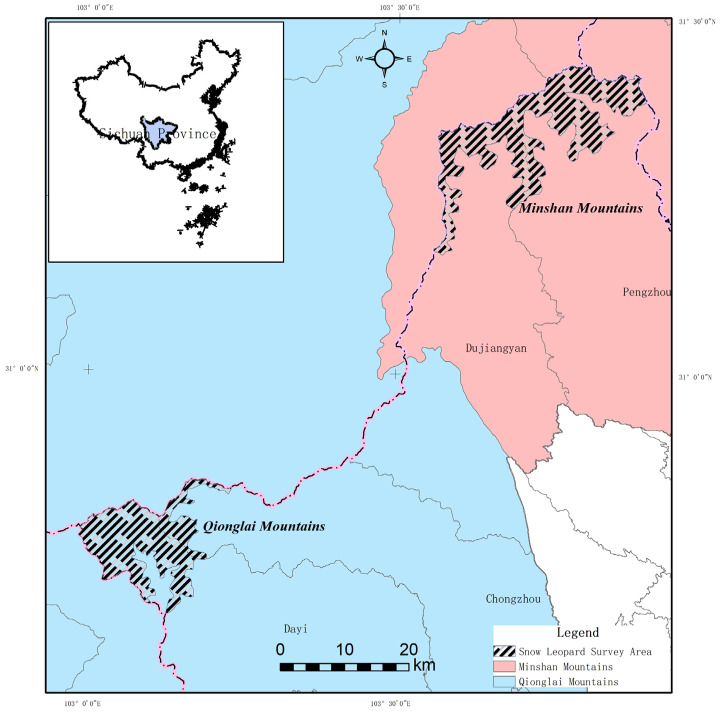
Overview of the Study Area.

The Chengdu section of the Giant Panda National Park is located in the northwestern part of the Chengdu Plain. It covers an area of 1459.48 km^2^, falling within the administrative jurisdictions of Dujiangyan City, Pengzhou City, Chongzhou City and Dayi County. The area straddles two major mountain systems: the Qionglai Mountains and the Min Mountains. The terrain is predominantly characterized by mid-mountain, deeply incised topography, with general elevations sloping from higher in the northwest to lower in the southeast. Slopes are typically steep, ranging from 30 to 45 degrees. The highest point reaches an altitude of 3868 m, while the lowest point is at 1638 m. The geological structure is complex, primarily consisting of limestone, sandstone, conglomerate, and granite, with soils exhibiting a distinct vertical zonation pattern. The climate is classified as a mid-subtropical humid monsoon mountain type, with a mean annual temperature of 12.3 °C and an average annual precipitation ranging from 1300 to 1450 mm. This region also serves as a crucial corridor for gene flow between giant panda populations in the Min and Qionglai mountain systems (Figure 1) [24,25].

### 2.2. Study Methods

#### 2.2.1. Snow Leopard Occurrence Data

Snow leopard occurrence data were collected through an analysis of recent infrared camera survey data and routine patrol monitoring records within the Chengdu section. Building upon historical distribution records, this study focused on areas above 2800 m elevation where snow leopard activity was considered possible. The total area of these potential activity zones was approximately 436.98 km^2^. This zone was subdivided into two survey regions: the Min Mountains survey region (covering Pengzhou and Dujiangyan, area: 254.73 km^2^) and the Qionglai Mountains survey region (covering Dayi and Chongzhou, area: 182.5 km^2^). Within the survey area, a grid system with 10 km^2^ cells was established as the fundamental survey unit, resulting in a total of 48 grid units. In the Qionglai Mountains survey region, 2–3 infrared cameras were deployed per grid unit (In cases where there are more than two cameras, they are also placed at the same survey site. The purpose of having multiple cameras is to capture images of snow leopards from different angles, thereby facilitating individual identification), while in the Min Mountains survey region, 1 camera was deployed per unit. A total of 89 infrared cameras were installed across the study area from 2021 to 2023 (Table 1). Considering the challenges associated with snow leopard habitats—characterized by high altitude, remoteness, and harsh climate—the survey methodology was supplemented by line transect surveys and interviews to obtain additional occurrence data. Transects were established at an intensity of one transect per 10 km^2^ grid, with each transect not exceeding 5 km in length. Surveyors recorded all signs of snow leopard presence, including live sightings, carcasses, scats, hair, and footprints, during transect walks.

Following the division of the survey area into sampling units based on mountain ranges and watersheds, interviews were conducted with personnel engaged in long-term patrol monitoring within the study area or with residents of nearby communities. Interviewees were asked whether they had observed snow leopards or their signs within specific sampling units, and their responses were recorded. At least one informant was ensured for each sampling unit. A total of 49 preliminary snow leopard occurrence points were collected. Given the species’ extensive home range, occurrence points located too closely may introduce spatial autocorrelation, which can reduce the predictive accuracy of the model [26]. Furthermore, existing research indicates that the activity radius of snow leopards in mountainous regions is approximately 6.28–8.13 km [27]. Therefore, a distance threshold of 6 km was set for this study. Redundant occurrence points with a separation distance less than this threshold were eliminated. Ultimately, 13 spatially independent snow leopard coordinate points were retained for subsequent modeling.

#### 2.2.2. Environmental Covariate Data

This study selected climatic, topographic, vegetation, and anthropogenic disturbance factors that influence snow leopard survival for subsequent analysis. To prevent overfitting caused by original environmental covariates and ensure the accuracy of model predictions, all required covariates were standardized and resampled to a 2.5 min resolution using the bilinear interpolation method [28]. Furthermore, to mitigate multicollinearity, covariates exhibiting a high degree of correlation (|r| ≥ 0.80) were identified and excluded based on a Pearson correlation coefficient matrix. Finally, we selected 15 variables, such as land use and climate, among which the correlation between DEM and EVP was 0.9. Because these were important indicators for predicting suitable snow leopard habitat, which included in the analysis (Appendix A).

From an initial set of 39 environmental covariates, 15 key indicators were selected for the final analysis. These include: eight climatic variables—Annual Evaporation (EVP), Annual Ground Soil Temperature (GST), Annual Precipitation (PRE), Annual Average Atmospheric Pressure (PRS), Annual Average Relative Humidity (RHU), Annual Sunshine Duration (SSD), Annual Average Temperature (TEM), and Annual Average Wind Speed (WIN); two topographic variables derived from the Digital Elevation Model (DEM)—the DEM itself and River distribution; one vegetation variable—the Normalized Difference Vegetation Index (NDVI); and four anthropogenic disturbance variables—distance to Human Settlements (SET), distance to Tourism sites (TOU), Land Use type (LAN), and distance to Roads (Table 2).

#### 2.2.3. Species Distribution Modeling

The Maximum Entropy Model (MaxEnt), the software version is 3.3.4, and the source of the software is https://biodiversityinformatics.amnh.org/open_source/maxent/ (accessed on 1 June 2025), a widely utilized and robust model for predicting species habitat suitability, demonstrates particularly reliable predictive accuracy even with limited occurrence data [29]. This study employed the MaxEnt model to analyze the spatial distribution patterns of snow leopard habitats and their influencing factors. The filtered snow leopard occurrence points and environmental covariates were imported into MaxEnt software. Due to the small sample size in this calculation, in order to reduce noise, instead of using the default 10,000 background points, the background point parameter was modified to 1000. We set the regularization multiplier to 2.0. The data were randomly partitioned, with 75% used for model training and the remaining 25% reserved for model testing. Model performance was evaluated using the Area Under the Receiver Operating Characteristic Curve (AUC) based on 10 bootstrap iterations. An AUC value >0.8 indicates good model performance, while a value >0.9 signifies excellent performance. The relative contribution of environmental covariates was assessed using the Jackknife test. The classification threshold for distinguishing suitable habitat was determined using the “Maximum training sensitivity plus specificity” logistic threshold. Subsequently, areas with habitat suitability values above this threshold were classified as suitable or potential snow leopard habitat using the reclassification function in ArcGIS (v10.8). These classification results were then calibrated against field survey data. Finally, the quality grades of the regional snow leopard habitats were determined based on a standard habitat evaluation procedure.

## 3. Results

### 3.1. Snow Leopard Distribution Range

Based on the field surveys, a total of 49 signs of snow leopard activity were recorded. Among these, 46 signs were located within the predefined survey area, covering 13 survey grids. Line transect surveys accounted for 39 activity signs distributed across 9 grids, while infrared camera monitoring captured snow leopard images at 11 camera locations, covering 6 grids. Among the four surveyed regions, the Dayi area yielded the highest number of snow leopard signs (44 signs), covering 11 grids. This was followed by the Pengzhou area with 4 signs across 2 grids. The Chongzhou area had the fewest signs, with only 1 sign recorded in 1 grid. No snow leopard activity signs were detected in the Dujiangyan area (Table 3).

### 3.2. Model Performance

**Figure 2:**
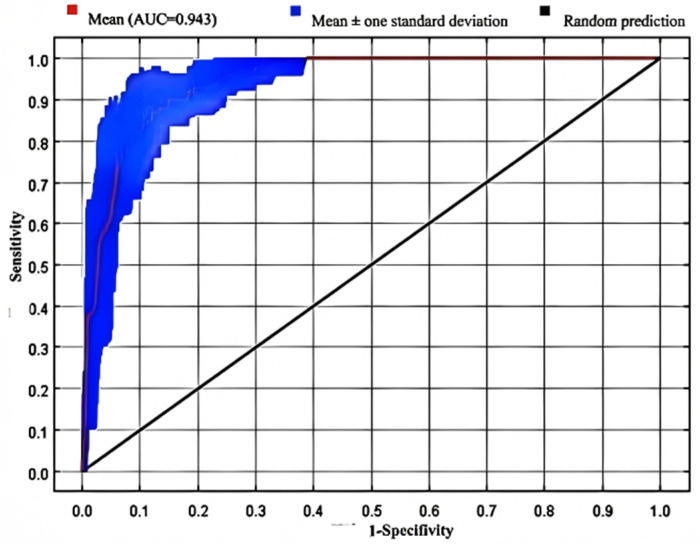
The Receiver Operating Characteristic (ROC) Validation Curve of Snow Leopard Habitat Prediction Results.

The accuracy evaluation of the MaxEnt model showed that the mean AUC value for the Receiver Operating Characteristic (ROC) curve reached 0.943 (Figure 2), indicating an excellent level of predictive performance. This confirms that the model reliably reflects the actual distribution of the snow leopard population in the study area.

### 3.3. Contribution of Environmental Variables

The geographical distribution of animals is governed by multiple factors, while habitat quality is modulated by diverse environmental variables [30]. According to the results of the MaxEnt model, different environmental variables contributed significantly and variably to the predicted species distribution. Mean TEM, Mean GST, and SET were identified as the core factors affecting snow leopard habitat suitability. Among these, Mean GST had a permutation importance of 41.4%, far exceeding that of other variables, demonstrating its decisive role in predicting snow leopard habitat (Table 4).

Specifically, the contribution rates of mean TEM and mean GST were 27% and 17.3%, respectively, indicating these two climatic factors dominate the assessment of habitat suitability for snow leopards. Concurrently, the distribution of snow leopards in the Chengdu section was also significantly influenced by the proximity to SET. Furthermore, the NDVI and PRE also had notable impacts on habitat suitability, with contribution rates reaching 10.2% and 7.5%, respectively. This underscores the crucial supporting role of vegetation cover and water availability in snow leopard habitat selection. TOU and LAN similarly exerted a certain degree of influence on habitat suitability, likely due to habitat fragmentation and environmental changes induced by human activities. The influence of the remaining variables on snow leopard habitat suitability was minimal.

**Figure 3:**
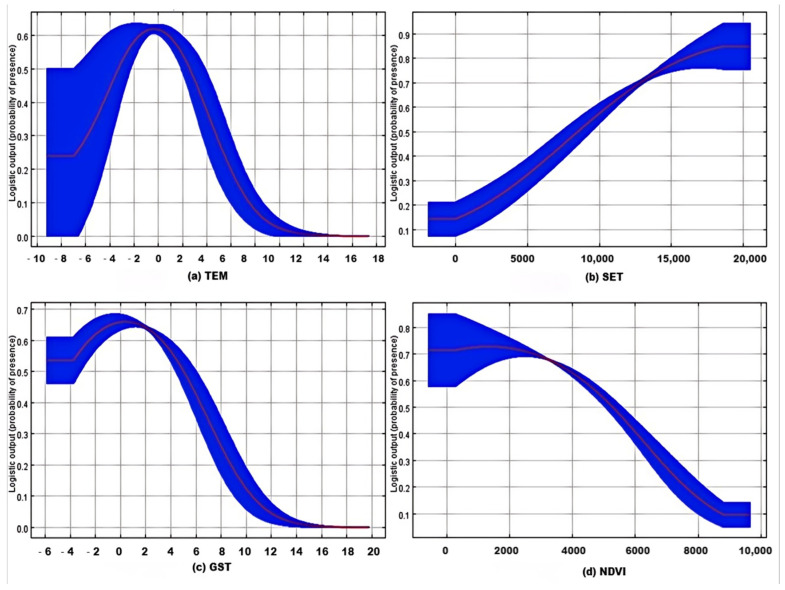
Single Response Curve Analysis of Key Variables. (**a**) Effect of mean Annual Average Temperature (TEM) on the probability of species presence; (**b**) Effect of Human Settlements (SET) on the probability of species presence; (**c**) Effect of mean Annual Ground Soil Temperature (GST) on the probability of species presence; (**d**) Effect of the Normalized Difference Vegetation Index (NDVI) on the probability of species presence.

Analysis of single-variable response curves revealed distinct habitat utilization characteristics of the snow leopard (Figure 3). The species reached peak suitability in low-temperature environments, specifically at a mean annual air temperature of approximately −2 °C (with a probability peak of 0.6) and a mean annual soil temperature of about −1 °C (with a probability peak of 0.65). A deviation from these optimal temperatures, either higher or lower, led to a sharp decline in predicted occurrence probability, with the decline being more pronounced when temperatures exceeded the optimal range. Furthermore, the probability of snow leopard presence showed a continuous negative correlation with distance from human settlements. The probability began to increase significantly when the distance exceeded approximately 5 km, approaching its peak probability (around 0.9) at a distance of about 17.5 km, indicating a strong tendency for snow leopards to avoid areas of human activity. In contrast, vegetation cover exhibited a significant negative effect. Snow leopards showed a clear preference for sparse vegetation environments, such as alpine meadows or bare rock, corresponding to an NDVI value of around 2000 (probability ~0.7). The probability dropped sharply to below 0.1 when the NDVI increased to 8000, representing dense vegetation.

### 3.4. Habitat Suitability Distribution

#### 3.4.1. Habitat Suitability Assessment for the Study Area

Based on the Maximum training sensitivity plus specificity logistic threshold and the TPT balance threshold derived from the MaxEnt model output, the predicted snow leopard habitat was reclassified. This process facilitated the assessment of habitat quality within the study area, ultimately generating a snow leopard habitat distribution map.

According to the MaxEnt model evaluation, the total area of snow leopard habitat within the entire assessment region was 876.92 km^2^. Prime habitat and moderately suitable habitat constitute a mere 14.41% and 14.42% of the total assessment area, respectively. These findings reveal that snow leopard habitat across the entire evaluation region is critically limited.

#### 3.4.2. Snow Leopard Habitat Suitability in the Chengdu Section

**Figure 4:**
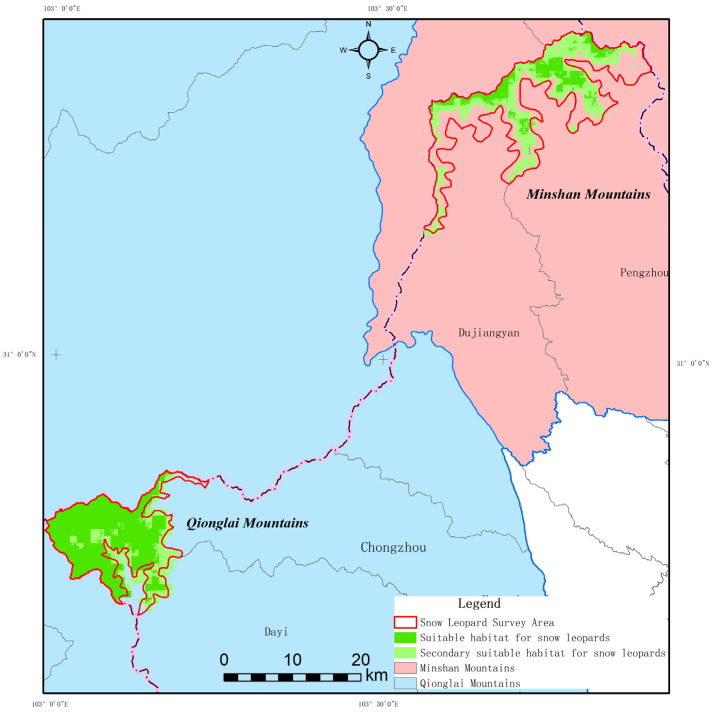
Snow Leopard Habitat Suitability Distribution Map in the Chengdu Area.

MaxEnt model evaluation shows snow leopard habitat within surveyed zones totals 320.98 km^2^ (Figure 4). While overall habitat remains limited, distribution varies by sub-region. The Dayi area, with highest snow leopard sign density, exhibits concentrated, larger suitable/moderately suitable habitat, linked to its unique geographic-ecological advantages: abundant water, favorable microclimate, and lower human disturbance, collectively supporting these elusive cats.

A detailed breakdown by administrative section revealed the following patterns (Table 5): In the Chongzhou area, the total snow leopard habitat area was 29.21 km^2^, accounting for 86.42% of its alpine ecosystem area. This included 18.83 km^2^ (55.71%) of suitable habitat and 10.38 km^2^ (30.71%) of moderately suitable habitat, indicating an overall medium level of habitat quality. The Dayi area possessed the most extensive snow leopard habitat within its alpine ecosystem, totaling 144.54 km^2^, which represented 97.37% of the area’s alpine zone. This comprised 119.66 km^2^ (80.61%) of suitable habitat and 24.88 km^2^ (15.41%) of moderately suitable habitat, demonstrating superior habitat quality. In contrast, the Dujiangyan area had a snow leopard habitat area of 47.32 km^2^ within its alpine ecosystem, constituting 49.03%. This included 10.08 km^2^ (10.44%) of suitable habitat and 34.24 km^2^ (35.47%) of moderately suitable habitat, reflecting relatively limited habitat quality. The Pengzhou area contained a total snow leopard habitat area of 99.91 km^2^, accounting for 63.15%. This consisted of 29.10 km^2^ (18.39%) of suitable habitat and 70.81 km^2^ (44.76%) of moderately suitable habitat, indicating an overall lower level of habitat quality.

## 4. Discussion

As an excellent tool for predicting species distribution, the MaxEnt model has been widely validated in numerous studies for its superior stability [31,32]. Studying the habitat of the snow leopard—often referred to as the “ghost of the mountains”—poses significant challenges. Its habitats is typically located in high-altitude, remote areas with harsh climatic conditions and minimal human presence. These factors make data collection extremely difficult, often resulting in sparse or incomplete sample data. Under such conditions of limited data, the MaxEnt model demonstrates unique advantages over other methods, as it can more effectively utilize scarce information to achieve high-precision predictions [33]. This model excels at integrating multiple environmental variables within complex ecosystems, and the comprehensiveness of these variables is key to achieving high-quality predictions [34]. The MaxEnt model constructed in this study performed excellently, with an AUC value >0.9, indicating highly reliable prediction results [35]. These predictions were further effectively validated by field observation data. Infrared camera images of snow leopard activity showed a high degree of concordance between the model-predicted areas of high habitat suitability and the actual range of snow leopard activity. Survey data revealed that snow leopard images were captured most frequently by infrared cameras in the Dayi area (a total of 11 image points across 6 survey grids), while the number of images from the Pengzhou and Chongzhou areas was significantly lower. This directly reflects the superior habitat quality in Dayi, which can provide snow leopards with more extensive living space and richer resources, thereby supporting higher activity frequency. This field observation result is highly consistent with the MaxEnt model’s prediction that the core area of high suitability habitat is primarily distributed in the Dayi area.

This study not only validated model accuracy but also provided deeper insights into the core mechanisms driving snow leopard habitat selection in the Chengdu section. The MaxEnt model results indicated that mean GST was the decisive factor influencing snow leopard distribution (with a permutation importance as high as 41.4%), its contribution significantly surpassing that of mean TEM, NDVI, and DEM [17,18,36]. This reflects the spatiotemporal heterogeneity inherent in the habitats of specific wildlife across different regions [37]. The response curve indicated that the optimal habitat for snow leopards in the Chengdu section occurred in environments with a GST of approximately −1 °C (peak probability of presence reaching 0.65), which aligns well with their adaptation to low temperatures originating from the Qinghai–Tibet Plateau [38]. Furthermore, the probability of snow leopard presence reached 0.9 at a distance of about 17.5 km from SET, a result consistent with the “human avoidance behavior” observed in regions such as Wolong and the Qilian Mountains [15,18]. The combined effect of these driving factors has resulted in a highly uneven spatial pattern of snow leopard habitat within the Chengdu section. The Dayi area concentrated up to 88% of all recorded snow leopard signs, with 97.4% of its alpine ecosystem area assessed as suitable or moderately suitable habitat. This distribution pattern is similar to that of the core zone in Wolong Nature Reserve (81% suitable habitat) [18]. In contrast, the proportion of suitable habitat in areas like Pengzhou and Dujiangyan was less than 20%. This stark disparity establishes the critical status of the Dayi area as the core conservation unit for snow leopards in the region. However, compared to snow leopard habitats in the Himalayan Mountains and the Three-River-Source region [39,40], the total snow leopard habitat area in the Chengdu section (320.98 km^2^) is notably smaller and exhibits a higher degree of fragmentation, underscoring the urgent need for targeted conservation measures.

During the survey, two additional disturbance factors, yak and horse, were documented within the Chengdu section. Yaks were recorded most frequently (235 occurrences), while horses were recorded only 4 times. Notably, these disturbances were primarily concentrated in the Dayi area. Previous studies have indicated that yaks can also serve as a prey source for snow leopards [41], which may partially explain the presence of snow leopards in the Dayi area compared to their absence in the adjacent Chongzhou area. Consequently, it is recommended to enhance cross-regional collaborative management. Provincial or national authorities should coordinate synchronized snow leopard survey and monitoring efforts across Wenchuan, Maoxian, and neighboring protected areas to obtain accurate population data for informed decision-making. A systematic assessment of the dynamics of snow leopard prey resources is imperative. Specialized surveys should investigate the distribution and density changes in natural prey species, such as blue sheep and pikas, as well as yaks in the Dayi and Chongzhou areas, to ensure the integrity of the snow leopard’s ecological foundation. Furthermore, grazing activities require scientific management, and their net effects warrant in-depth investigation [42]. Building upon strengthened oversight and the control of grazing scope and intensity to mitigate competitive interference, it is essential to systematically quantify the comprehensive impact of yaks—functioning as both a potential food source and a disturbance factor—on snow leopard population dynamics (i.e., the net effect). This will provide a scientific basis for formulating refined management policies that balance snow leopard conservation with the livelihoods of local herders.

## 5. Conclusions

This study integrated field survey methods, including infrared camera monitoring, with MaxEnt modeling to assess snow leopard habitat in the Chengdu section of the Giant Panda National Park. The results revealed a distinct “higher in the west, lower in the east” spatial pattern in habitat distribution, with the core distribution area highly concentrated in the alpine regions of Dayi County. Environmental temperature, particularly soil temperature, was identified as the dominant factor determining habitat suitability, confirming the species’ strong reliance on cold environments. Snow leopards exhibited a clear preference for sparse vegetation habitats and were significantly affected by human disturbance. Currently, suitable habitat within the section is scarce and unevenly distributed, with high-quality core areas primarily located in the western alpine zones of Dayi County. Consequently, there is an urgent need to prioritize conservation efforts and enhance ecological connectivity within this core area, while strictly controlling the expansion of human activities based on settlement density thresholds. These findings not only fill a critical knowledge gap regarding snow leopard habitat assessment in this region but also provide a vital scientific foundation for functional zoning within protected areas, habitat conservation, and the development of disturbance management strategies.

## Figures and Tables

**Table 1:** Infrared Camera Monitoring Grid Units and Monitoring Duration.

District (County)	Number of Grid Units	Number of Infrared Cameras	Effective Working Days
Dujiangyan	12	12	113–125
Chongzhou	7	20	110–121
Dayi	14	42	128–159
Pengzhou	15	15	112–137

**Table 2:** Types of Environmental Covariates and Their Sources.

Factor Type	Variable	Data Sources
Climate	EVP	WorldClim (https://www.worldclim.org/) (accessed on 8 June 2025)
	GST	
	PRE	
	PRS	
	RHU	
	SSD	
	TEM	
	WIN	
Topography	DEM	Geographic Information Monitoring Cloud Platform (http://www.dsac.cn/) (accessed on 9 June 2025)
	River	Using Arcgis Hydrological Analysis Tool to Extract DEM Data
Vegetation	NDVI	Resource and Environment Science Data Platform (http://www.resdc.cn/) (accessed on 10 June 2025)
Anthropogenic Disturbance	SET	NASA Socioeconomic Data and Applications Center (https://www.earthdata.nasa.gov/) (accessed on 11 June 2025)
	TOU	
	LAN	
	Distance to Roads (Road)	

**Table 3:** Table of Snow Leopard Distribution Locations.

District (County)	Sign/Grid	Feces	Infrared Cameras	Total
Dujiangyan	Sign	4	0	4
	Grid	2	0	2
Chongzhou	Sign	4	0	4
	Grid	2	0	2
Dayi	Sign	30	14	44
	Grid	7	5	10 *
Pengzhou	Sign	0	1	1
	Grid	0	1	1

* represents the removal of duplicate data, as it was found that there were overlaps between the grids where snow leopard feces were found and those where snow leopards were detected by infrared cameras.

**Table 4:** Analysis of Environmental Variables Contribution and Importance using the Cut-off Method.

Variable	Contribution Rate (%)	Importance of Displace
TEM	27	22.1
SET	19.5	11.4
GST	17.3	41.4
NDVI	10.2	6.6
PRE	7.5	2.1
TOU	6.2	6.2
LAN	3.1	3.7
Road	2.9	4.1
River	2	0.4
RHU	1.2	0
EVP	1	0
PRS	1	0
DEM	0.8	1.3
WIN	0.2	0.3
SSD	0	0.2

**Table 5:** Snow leopard habitat overview table in Chengdu area.

Area Name	Qionglai Mountain Area		Minshan Area		Total
Survey area/km^2^	182.25		254.73		436.98
District Name	Chongzhou	Dayi	Dujiangyan	Pengzhou	
Survey area/km^2^	33.80	148.45	96.52	158.21	
Habitat area/km^2^	29.21	144.54	47.32	99.91	320.98
Suitable habitat area/km^2^	18.83	119.66	13.08	29.10	180.66
Proportion of suitable habitat/%	55.70	80.60	13.55	18.40	100
Area of suboptimal habitat/km^2^	10.38	24.88	34.24	70.81	140.32
Proportion of suboptimal habitat/%	30.72	16.76	35.48	44.76	100

## Data Availability

Data will be made available upon request.

## Appendix A

**Table A1:** Environment variable correlation matrix.

	DEM	EVP	GST	SET	TOU	NDVI	PRE	PRS	RIVER	ROAD	RUH	SSD	LAN	TEM	WIN
DEM	1	−0.96	−0.96	0.54	0.61	−0.63	0.54	−0.96	0.3	0.34	0.74	0.89	0.21	−0.96	0.95
EVP	−0.96	1	0.99	−0.53	−0.6	0.63	−0.59	1	−0.23	−0.32	−0.8	−0.9	−0.2	1	−0.99
GST	−0.96	0.99	1	−0.53	−0.58	0.63	−0.58	0.99	−0.23	−0.36	−0.79	−0.9	−0.21	1	−0.99
SET	0.54	−0.53	−0.53	1	0.52	−0.45	0.58	−0.53	0.09	0.22	0.6	0.42	0.22	−0.53	0.5
TOU	0.61	−0.6	−0.58	0.52	1	−0.43	0.33	−0.6	0.09	0.22	0.41	0.68	0.1	−0.58	0.55
NDVI	−0.63	0.63	0.63	−0.45	−0.43	1	−0.36	0.63	−0.14	−0.29	−0.48	−0.58	−0.17	0.63	−0.62
PRE	0.54	−0.59	−0.58	0.58	0.33	−0.36	1	−0.55	0.22	−0.02	0.94	0.29	0.18	−0.59	0.54
PRS	−0.96	1	0.99	−0.53	−0.6	0.63	−0.55	1	−0.22	−0.35	−0.77	−0.92	−0.2	1	−0.99
RIVER	0.3	−0.23	−0.23	0.09	0.09	−0.14	0.22	−0.22	1	0.05	0.25	0.16	0.05	−0.23	0.22
ROAD	0.34	−0.32	−0.36	0.22	0.22	−0.29	−0.02	−0.35	0.05	1	0.09	0.31	0.12	−0.35	0.4
RUH	0.74	−0.8	−0.79	0.6	0.41	−0.48	0.94	−0.77	0.25	0.09	1	0.52	0.21	−0.8	0.76
SSD	0.89	−0.9	−0.9	0.42	0.68	−0.58	0.29	−0.92	0.16	0.31	0.52	1	0.15	−0.89	0.88
LAN	0.21	−0.2	−0.21	0.22	0.1	−0.17	0.18	−0.2	0.05	0.12	0.21	0.15	1	−0.21	0.21
TEM	−0.96	1	1	−0.53	−0.58	0.63	−0.59	1	−0.23	−0.35	−0.8	−0.89	−0.21	1	−0.99
WIN	0.95	−0.99	−0.99	0.5	0.55	−0.62	0.54	−0.99	0.22	0.4	0.76	0.88	0.21	−0.99	1

Notes: Annual Evaporation (EVP), Annual Ground Soil Temperature (GST), Annual Precipitation (PRE), Annual Average Atmospheric Pressure (PRS), Annual Average Relative Humidity (RHU), Annual Sunshine Duration (SSD), Annual Average Temperature (TEM), Annual Average Wind Speed (WIN); Digital Elevation Model (DEM), River, Normalized Difference Vegetation Index (NDVI); distance to Human Settlements (SET), distance to Tourism sites (TOU), Land Use type (LAN), and distance to Roads.
